# Epistaxis und antithrombotische Medikation: eine Analyse der Daten einer gesetzlichen Krankenversicherung in Niedersachsen

**DOI:** 10.1007/s00106-020-00940-y

**Published:** 2020-09-14

**Authors:** A. E. Althaus, U. Arendt, F. Hoffmann, J. Lüske, M. H. Freitag, K. Jobski, M. Dörks

**Affiliations:** 1 Department für Versorgungsforschung, Abteilung Allgemeinmedizin, Carl von Ossietzky Universität Oldenburg, Oldenburg, Deutschland; 2 Department für Versorgungsforschung, Abteilung Ambulante Versorgung und Pharmakoepidemiologie, Carl von Ossietzky Universität Oldenburg, Oldenburg, Deutschland; 3 Praxis Dr. Lüske, Oldenburg, Deutschland; 4 Theaterwall 43, 26122 Oldenburg, Deutschland

**Keywords:** Epistaxis, Nasenbluten, Antikoagulation, NOAK, Prävalenz, Epistaxis, Nosebleed, Anticoagulation, NOAC, Prevalence

## Abstract

**Hintergrund:**

Die Epistaxis ist ein häufiges Symptom in der Arztpraxis und assoziiert mit verschiedenen Komorbiditäten und Medikamenten, insbesondere Antikoagulanzien. Trotz ihrer Alltäglichkeit gibt es nur wenige Daten zur Häufigkeit ihres Auftretens und möglichen Risikofaktoren.

**Methoden:**

Die Studie untersuchte anhand einer großen Patientenpopulation (AOK Niedersachsen) über 10 Jahre (2007–2016) die Versorgung von Epistaxis in Niedersachsen. Alter bei Diagnose, Begleitmedikation und Komorbiditäten wurden analysiert und die Prävalenz dargestellt.

**Ergebnisse:**

162.167 Versicherte der AOK Niedersachen wurden zwischen 2007 und 2016 aufgrund einer Epistaxis in 308.947 Fällen ärztlich vorstellig. Die meisten Patienten wurden ausschließlich ambulant behandelt (96,6 %). Über den Studienzeitraum stieg die Prävalenz um 21 % (Anstieg von 8,7 auf 9,3 pro 1000 Versicherte/Jahr) bei stabiler Prävalenz für stationäre Vorstellungen (0,2 pro 1000 Versicherte/Jahr). Die höchsten Prävalenzen fanden sich bei Personen bis 20 und über 80 Jahre. In 17,5 % aller Epistaxisfälle wurden Antithrombotika erfasst (9,5 % orale Antikoagulanzien). Über den Studienzeitraum konnte eine erhöhte Verschreibung von Antikoagulanzien (7,7 % in 2007 auf 11,8 % in 2016, insbesondere NOAK) dokumentiert werden.

**Schlussfolgerung:**

Neben der arteriellen Hypertonie, dem männlichen Geschlecht sowie der typischen Altersverteilung bestand auch häufig eine Medikation mit Antikoagulanzien. Über den untersuchten Zeitraum zeigte sich eine Zunahme von Epistaxisfällen bei gleichzeitig ansteigender Verschreibungshäufigkeit von NOAK, nicht jedoch von schweren hospitalisationsbedürftigen Epistaxisfällen. Eine abschließende Beurteilung hinsichtlich eines möglichen kausalen Zusammenhangs muss in weiteren Studien untersucht werden.

## Abstract (en)

**Background:**

Epistaxis is a common symptom in the medical practice. It is associated with various comorbidities and the use of medications, especially anticoagulants. Despite the high lifetime prevalence, there is limited data on prevalence and possible risk factors.

**Methods:**

The study examines epistaxis care in a large patient population (AOK Lower Saxony) over a ten-year period (2007–2016). Risk factors, age at diagnosis, concomitant medication and comorbidities were analysed and the prevalence in the study period calculated.

**Results:**

162,167 patients visited their doctors between 2007–2016 (308,947 cases). Most patients were treated as outpatients (96.6%) and 54% of patients were men. Over the study period, the prevalence of epistaxis rose by 21% (increase from 8.6 to 9.3 per 1000 insured persons per year) with a comparatively stable prevalence for the inpatient setting (0.2 per 1000 insured persons per year). In 54,105 of all epistaxis cases (17.5%), the use of antithrombotic drugs was recorded (oral anticoagulants: 9.5%). During the study period, increased prescribing of oral anticoagulants (from 7.7% of cases in 2007 to 11.8% in 2016), especially of NOAC was documented (from 0.1% of cases in 2011 to 5.1% in 2016).

**Conclusion:**

In addition to arterial hypertension, the predominant male sex and the typical age distribution, we found that cases of epistaxis often received anticoagulation therapy. This study showed an increase of epistaxis along with rising prescriptions of NOAC. In contrast, no increase of severe epistaxis cases leading to hospitalization was found.

## Hintergrund

Die Epistaxis beschreibt unterschiedliche Formen des nasalen Blutverlusts. Trotz ihrer Alltäglichkeit gibt es zur Häufigkeit ihres Auftretens nur wenige Daten. In einer kleinen Befragung englischer Patienten berichteten etwa 60 % der Befragten von mindestens einer Episode während ihrer Lebenszeit, nur jeder Zehnte nahm allerdings deswegen das Gesundheitswesen in Anspruch [1].

Das Auftreten in Abhängigkeit vom Alter wird als zweigipflig beschrieben – mit einem Peak bei Kindern und älteren Patienten ab dem 60. Lebensjahr – und gilt als eng verknüpft mit verschiedenen Komorbiditäten und der Einnahme von Medikamenten, insbesondere Thrombozytenaggregations- und Gerinnungshemmern [2–4]. Außerdem wird ein saisonal gehäuftes Auftreten vermutet. Männer sind häufiger betroffen als Frauen [4].

Der Schweregrad bzw. das klinische Erscheinungsbild reicht vom spontan sistierenden Nasenbluten beim Infekt der oberen Atemwege bis hin zum lebensbedrohlichen Blutverlust bei einer akuten fulminanten Epistaxis. Ursächlich können viele Faktoren sein. So kann sie z. B. unter einer Antikoagulanzientherapie oder als Zeichen einer Tumorerkrankung im Nasennebenhöhlensystem auftreten. Chronisch bzw. rezidivierend (z. B. bei M. Osler) kann sie eine Anämie (mit)verursachen. Da die Epistaxis häufig ohne Therapie sistiert, besteht die Annahme, dass der Großteil der Epistaxisepisoden von Patienten selbst behandelt wird. [1, 4, 5].

In der HNO-Heilkunde – hier stellt die Epistaxis den häufigsten Notfall dar [6] – wird zwischen anteriorer bzw. posteriorer Epistaxis unterschieden. Erstere tritt vornehmlich im Bereich des Locus Kiesselbachi auf, und dieser ist mit 90–95 % der Fälle auch die häufigste Lokalisation der Epistaxis [7, 8]. Sie ist in ca. 65 % der Fälle durch Allgemeinmaßnahmen wie die Kompression des anterioren Septums und die Applikation abschwellender Nasentropfen leicht beherrschbar bzw. ambulant haus- oder kinderärztlich therapierbar [9]. Im Gegensatz dazu nimmt die posteriore Epistaxis häufig einen fulminanteren Verlauf und muss in ca. 1 % aller Fälle operativ versorgt werden [2, 10].

Der gerinnungshemmende Effekt der neuen oralen Antikoagulanzien (NOAK, auch als nicht-Vitamin-K-abhängige oder direkte orale Antikoagulanzien bezeichnet) beruht, anders als bei Heparinen und Vitamin-K-Antagonisten, auf der direkten Hemmung der Blutgerinnungsfaktoren. Seit dem Jahr 2012 werden in Deutschland steigende Verordnungszahlen von NOAK mitgeteilt [11–13].

Obwohl die Epistaxis ein häufig auftretendes Symptom darstellt, existieren bisher keine Daten zur ambulanten Versorgung von Patienten mit Epistaxis in Deutschland. Vor allem die Frage, wie die Prävalenz der Epistaxis durch die Veränderungen der demografischen Entwicklung und Verschreibungshäufigkeit von Antikoagulanzien – insbesondere der Einführung der NOAK ab dem Jahr 2008 – beeinflusst wurde, wollten wir anhand einer Sekundärdatenanalyse untersuchen. Ein weiterer Fokus lag auf der Charakterisierung der Epistaxisfälle hinsichtlich möglicher Einflussfaktoren für die Entwicklung einer Epistaxis.

## Methoden

Im Jahr 2016 waren 2,5 Mio. Versicherte Mitglied der AOK Niedersachsen, was etwa 36 % aller gesetzlich Versicherten in Niedersachsen entspricht [14]. Im Rahmen dieser Studie wurden anonymisierte Abrechnungsdaten der AOK Niedersachsen von Versicherten analysiert, für die eine gesicherte ambulante Diagnose oder stationäre Hauptentlassungsdiagnose der Epistaxis (R04 gemäß ICD-10, 10. Revision) in den Jahren 2007–2016 identifiziert wurde.

Für alle in die Studie eingeschlossenen Versicherten lagen neben demografischen Daten wie Alter und Geschlecht Informationen über quartalsweise vorliegende ambulante und exakt datierte stationäre Diagnosen vor. Zur besseren Untersuchung des Risikofaktors „Alter“ erfolgte eine Einteilung der Versicherten in Altersgruppen („≤10 Jahre“, „11–20 Jahre“, „21–40 Jahre“, „41–60 Jahre“, „61–80 Jahre“, „>80 Jahre“).

Ambulante Fälle wurden dem Quartal der Diagnosestellung zugeordnet, Personen konnten pro Quartal mehrmals als Fall diagnostiziert werden (verschiedene Fallnummern). Stationäre Fälle wurden mit dem zugehörigen Krankenhausaufnahmedatum datiert.

Des Weiteren enthielten die Daten Informationen zu Arzneiverordnungen antithrombotischer Mittel (B01 nach dem anatomisch-therapeutisch-chemischen Klassifikationssystem, ATC). Für jede Verordnung wurde die Dauer (Reichweite) anhand der Zahl der verordneten definierten Tagesdosen (DDD), beginnend mit dem Abgabedatum, bestimmt. Hiernach wurde ein Versicherter als mit einem antithrombotischen Arzneimittel therapiert angesehen, wenn er oder sie an mindestens einem Tag im Quartal der Epistaxisdiagnose entsprechende Medikation zur Verfügung hatte. Komorbiditäten wurden anhand der im gleichen Abrechnungsquartal kodierten Diagnosen aus dem ambulanten Bereich (Diagnosesicherheit „gesichert“) ermittelt.

Die Epistaxisprävalenz pro Jahr wurde pro 1000 Versicherte stratifiziert nach Alter ermittelt. Hierzu wurde die Zahl aller Versicherten mit mindestens einer Epistaxisdiagnose in dem entsprechenden Jahr bezogen auf die Anzahl aller Versicherten zur Jahresmitte erhoben. Weiter wurde das Auftreten hinsichtlich einer saisonalen Häufigkeit analysiert.

Die Datenanalyse erfolgte mit SAS (Version 9.4, SAS Institute Inc., Cary/NC, USA).

## Ergebnisse

### Epistaxisprävalenz im Studienzeitraum

**Figure 1:**
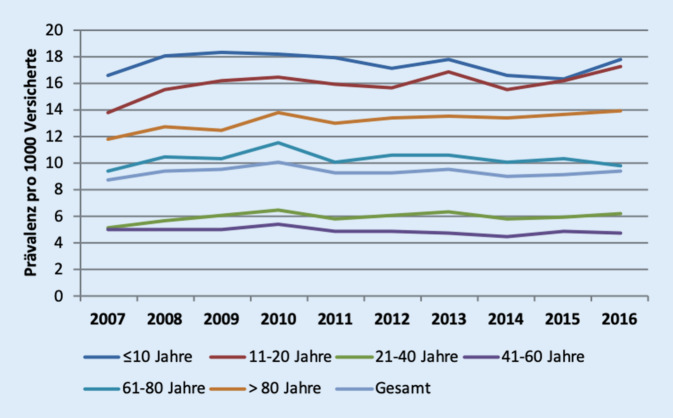

Insgesamt wurden 162.167 Versicherte der AOK Niedersachen zwischen 2007 und 2016 aufgrund einer Epistaxis in insgesamt 308.947 Fällen ärztlich vorstellig. Im Verlauf der Studie konnte ein Anstieg der Prävalenz von 8,7 (2007) auf 9,3 (2016) pro 1000 Versicherte dokumentiert werden (Abb. 1). Bezogen auf die Anzahl der Epistaxisfälle entspricht dies einer Zunahme von 21 % (27.093 Fälle im Jahr 2007 auf 32.872 Fälle im Jahr 2016). Die höchsten Prävalenzen fanden sich für die beiden jüngsten Altersgruppen, gefolgt von der Gruppe der über 80-jährigen Personen.

Für stationäre Fälle blieb die Prävalenz über die Jahre vergleichsweise stabil (0,2 pro 1000 Personen;* Anmerkung: nicht grafisch dargestellt*). Hier fand sich die höchste Prävalenz in der Altersgruppe der über 80-Jährigen (0,8 im Jahr 2007 bzw. 0,9 pro 1000 Personen im Jahr 2016).

### Patientencharakteristika

Das männliche Geschlecht machte 54 % aller Patienten aus (Tab. 1). Die meisten Patienten im Studienzeitraum wurden ausschließlich ambulant behandelt (96,6 %); insgesamt 5449 Patienten mussten aufgrund einer Epistaxis stationär aufgenommen werden, wobei die mediane Aufenthaltsdauer bei 4 Tagen lag.

**Table 1:** 

**Geschlecht**	
Männlich	87.601 (54,0 %)
Weiblich	74.566 (46,0 %)
**Ort der Vorstellung**	
Ambulant	156.718 (96,6 %)
Stationär	1204 (0,7 %)
Ambulant und stationär	4245 (2,6 %)
**Dauer der stationären Vorstellung**	
Median (Tage)	4 (2–5)
**Anzahl Vorstellungen während des Studienzeitraums**	
*1 Vorstellung während des Studienzeitraums*	98.184 (60,5 %)
*≥2 Vorstellungen während des Studienzeitraums*	63.983 (39,5 %)
Mehrere Vorstellungen im gleichen Quartal oder in 2 aufeinanderfolgenden Quartalen	48.989 (30,2 %)

Knapp 40 % der Patienten wurden mehrmals während des Studienzeitraums wegen einer Epistaxis vorstellig, bei über 75 % dieser Patienten erfolgten die Vorstellungen im gleichen Quartal oder in 2 aufeinanderfolgenden Quartalen.

### Fallcharakteristika

Entsprechend der Gesamtzahl der Patienten zeigten sich auch bezogen auf die Anzahl der Fälle Männer häufiger von Epistaxis betroffen (55,1 %). Das mediane Alter lag bei 47 Jahren (Interquartilsabstand, IQR: 16–72), wobei die stationären Patienten im Median 72 Jahre (IQR: 57–82) alt waren. Die Epistaxisfälle traten mit 53,8 % der Fälle häufiger in den Winterquartalen auf.

Bei 40,4 % der Fälle wurde eine arterielle Hypertonie diagnostiziert (bei 39,9 % der ambulanten und 68,7 % der stationären Fälle), bei 14,8 % eine chronische ischämische Herzkrankheit und bei 9,9 % Vorhofflimmern (ambulante Fälle: 9,9 %; stationäre Fälle: 26,7 %). Die Thrombozytopenie sowie der M. Osler lagen bei 0,9 % bzw. 0,4 % aller Epistaxisfälle vor.

In 54.105 aller Epistaxisfälle, entsprechend 17,5 %, wurde die Anwendung von Antithrombotika erfasst (9,5 % orale Antikoagulanzien). Insgesamt 8,1 % der Fälle hatten Vitamin-K-Antagonisten erhalten und 1,4 % NOAK. In 8,5 % der Epistaxisfälle wurde eine Therapie mit einem Thrombozytenaggregationshemmer registriert. Die Verschreibung von Heparin erfolgte nur bei 1,6 % der Fälle.

Beim stationären Patientenkollektiv wurde deutlich häufiger eine antithrombotische Therapie erfasst als bei den ambulant behandelten Epistaxisfällen (42,1 % bzw. 17,1 %) (Tab. 2).

**Table 2:** 

	Ambulant (*n* = 302.782)	Stationär (*n* = 6165)	Gesamt (*n* = 308.947)
**Alter in Jahren**			
**Median (IQR)**	46 (16–72)	72 (57–82)	47 (16–72)
**Altersgruppe**			
≤10 Jahre	49.191 (16,3 %)	203 (3,3 %)	49.563 (16,0 %)
11–20 Jahre	53.701 (17,7 %)	215 (3,5 %)	53.916 (17,5 %)
21–40 Jahre	39.356 (13,0 %)	287 (4,7 %)	39.643 (12,8 %)
41–60 Jahre	45.101 (14,9 %)	1106 (17,9 %)	46.207 (15,0 %)
61–80 Jahre	82.140 (27,1 %)	2810 (45,6 %)	84.950 (27,5 %)
>80 Jahre	33.124 (10,9 %)	1544 (25,0 %)	34.668 (11,2 %)
**Geschlecht**			
Männlich	166.877 (55,1 %)	3375 (54,7 %)	170.252 (55,1 %)
Weiblich	135.905 (44,9 %)	2790 (45,3 %)	138.695 (44,9 %)
**Saisonale Verteilung**			
Sommer (2. + 3. Quartal)	140.137 (46,3 %)	2742 (44,5 %)	142.879 (46,2 %)
Winter (1. + 4. Quartal)	162.645 (53,7 %)	3423 (55,5 %)	166.068 (53,8 %)
**Komorbiditäten**			
Essenzielle (primäre) Hypertonie (I10-I15)	120.702 (39,9 %)	4233 (68,7 %)	124.935 (40,4 %)
Chronische ischämische Herzkrankheit/KHK (I25)	43.970 (14,5 %)	1892 (30,7 %)	45.862 (14,8 %)
Myokardinfarkt (I21, I25.2)	9463 (3,1 %)	429 (7,0 %)	9892 (3,2 %)
Vorhofflimmern/-flattern (I48)	28.857 (9,5 %)	1645 (26,7 %)	30.502 (9,9 %)
Hirninfarkt (I63, I64, I69.3, I69.4)	10.141 (3,4 %)	496 (8,1 %)	10.637 (3,4 %)
Atherosklerose der Extremitätenarterien (I70.2)	4458 (1,5 %)	204 (3,3 %)	4662 (1,5 %)
Venöse Thromboembolie (I26, I80.1, I80.2, I80.3, I80.9)	3668 (1,2 %)	150 (2,4 %)	3818 (1,2 %)
Thrombozytopenie (D69.4, D69.5, D69.6)	2531 (0,8 %)	103 (1,7 %)	2634 (0,9 %)
Hereditäre hämorrhagische Teleangiektasie, M. Osler (I78)	1004 (0,3 %)	96 (1,6 %)	1100 (0,4 %)
Myelodysplastisches Syndrom (D46)	772 (0,3 %)	50 (0,8 %)	822 (0,3 %)
Bösartige Neubildung des Nasopharynx, Nasenhöhle oder der Nasennebenhöhlen (C11, C30, C31)	285 (0,1 %)	14 (0,2 %)	299 (0,1 %)
**Anwendung von Antithrombotika**	51.508 (17,1 %)	2597 (42,1 %)	54.105 (17,5 %)
*Orale Antikoagulanzien (OAK)*	27.745 (9,2 %)	1550 (25,1 %)	29.295 (9,5 %)
Vitamin-K-Antagonisten (VKA, ATC: B01AA)	23.702 (7,8 %)	1361 (22,1 %)	25.063 (8,1 %)
NOAK (ATC: B01AF01, B01AE07, B01AF02, B01AX06)	4498 (1,5 %)	240 (3,9 %)	4738 (1,5 %)
*Thrombozytenaggregationshemmer (TAH, ATC: B01AC)*	25.105 (8,3 %)	1134 (18,4 %)	26.239 (8,5 %)
*OAK* *+* *TAH*	3063 (1,0 %)	216 (3,5 %)	3279 (1,1 %)
*Heparin (ATC: B01AB)*	4667 (1,5 %)	428 (6,9 %)	5095 (1,6 %)

*ATC* anatomisch-therapeutisch-chemisches Klassifikationssystem, *ICD* internationale statistische Klassifikation der Krankheiten und verwandter Gesundheitsprobleme (10. Revision), *IQR* Interquartilsabstand

**Figure 2:**
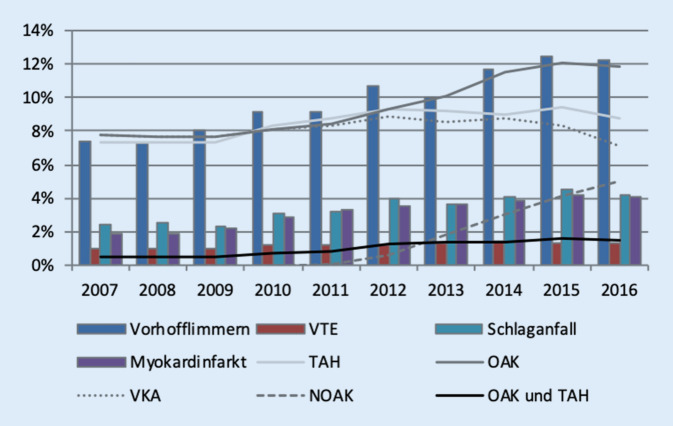

Während des Studienzeitraums stieg der Anteil der Fälle, die mit oralen Antikoagulanzien therapiert wurden von 7,7 % in 2007 auf 11,8 % in 2016. Während die Verschreibung von Vitamin-K-Antagonisten leicht abnahm (7,7 % in 2007 auf 7,1 % in 2016), stieg der Anteil von Epistaxisfällen, die eine Therapie mit einem NOAK erhielten, von 0,1 % in 2011 auf 5,1 % in 2016 (Abb. 2). Bei den Thrombozytenaggregationshemmern zeigte sich im Zeitverlauf ein Anstieg von 7,3 % in 2007 auf 8,7 % in 2016 mit einem Maximum von 9,3 % in 2012.

Zeitgleich konnte ein Anstieg von Vorhofflimmern (von 7,4 % im Jahr 2007 auf 12,2 % im Jahr 2016), Myokardinfarkt (1,9 auf 4,2 %) sowie Hirninfarkt (Apoplex) (2,4 auf 4,5 %) im Patientenkollektiv identifiziert werden (Abb. 2).

## Diskussion

In der vorliegenden Arbeit wurden erstmals anhand einer großen Patientenpopulation und über einen 10-Jahres-Zeitraum die Prävalenz der (ärztlich behandelten) Epistaxis sowie Charakteristika von Epistaxisfällen einschließlich ihrer Behandlung mit gerinnungshemmenden Arzneimitteln untersucht.

Der Hauptfokus der Arbeit lag hierbei auf der Frage, wie sich die Prävalenz der Epistaxis vor dem Hintergrund der Veränderungen der demografischen Entwicklung sowie der Verschreibungshäufigkeit von Antikoagulanzien – insbesondere der Einführung der NOAK ab dem Jahr 2008 – verändert hat.

### Prävalenz und Patientencharakteristika

Während des Studienzeitraums konnte eine Zunahme der Epistaxisprävalenz mit Arztkontakt von 21 % (Anstieg der Prävalenz von 8,7 auf 9,3 pro 1000 Personen) bei vergleichsweise gleich bleibender Prävalenz für stationäre Vorstellungen (0,2 pro 1000 Personen) – vereinbar mit Resultaten anderer Studien [3, 15] – dokumentiert werden.

Von den insgesamt 162.167 Patienten, die aufgrund einer Epistaxis in den Jahren 2007–2016 in 308.947 Fällen ärztlich vorstellig wurden, waren 55,1 % männlich. Auch Chaaban et al. berichten, dass 1,24-fach häufiger Männer diesbezüglich vorstellig wurden [16]. Auffallend war – wie auch bei Folz et al. – eine zweigipflige Altersverteilung mit der höchsten Prävalenz in den beiden jüngsten Altersgruppen und den über 80-jährigen Patienten [17].

Die vorliegende Studie lässt keine ätiologische Klärung zu. Aus anderen Studien ist bekannt, dass die Epistaxis bei Kindern durch lokale Manipulation (u. a. Nasenbohren, Fremdkörper), spielerische Verletzungen (z. B. Raufereien) und trockene Schleimhäute (z. B. im Rahmen von grippalen Infekten) gehäuft auftritt [18, 19]. Auch kann von einer niederschwelligen Vorstellungsrate aufgrund von Besorgnis der Eltern ausgegangen werden [20].

Weiter zeigte sich ein erhöhtes Auftreten in den Winterquartalen, was mit den Risikofaktoren trockene Luft- und schwankende Temperaturverhältnisse sowie den gehäuft auftretenden Erkältungsinfekten zusammenhängen könnte [21–23].

Die arterielle Hypertonie war die häufigste Komorbidität der Epistaxisfälle in diesem Versichertenkollektiv. Über ähnliche Ergebnisse berichten auch Weigel et al. sowie Kikidis et al. [4, 24]. In einer erst kürzlich erschienenen Studie von Kim et al. wurde die arterielle Hypertonie als mögliche Ursache der Epistaxis diskutiert [25].

Die Thrombozytopenie sowie der M. Osler waren – entsprechend ihrem natürlichen Auftreten – nur selten erfasste Komorbiditäten (0,9 % bzw. 0,4 %). Eine Assoziation zur Epistaxis ist bekannt [4].

Bei den stationären Fällen traten die untersuchten Erkrankungen deutlich häufiger auf als bei ambulant behandelten, ebenso wurde häufiger die Einnahme von Antikoagulanzien erfasst, was sicherlich auch auf das höhere Alter dieses Kollektivs zurückzuführen ist.

### Antithrombotische Therapie

Entsprechend den Resultaten von Weigel et al., Simmen et al. und Pollice et al. [4, 26, 27] wurde auch in unserem Kollektiv in rund 18 % der Epistaxisfälle die Anwendung von Antithrombotika (Vitamin-K-Antagonisten: 8,1 %) angegeben.

Parallel zum Anstieg der Epistaxisprävalenz konnte eine erhöhte Anzahl der Verschreibungen von Antikoagulanzien (7,7 % in 2007 auf 11,8 % in 2016) unter häufigerem Auftreten der indikationsgemäßen Diagnosen (Vorhofflimmern, Myokardinfarkt, Hirninfarkt) im Patientenkollektiv dokumentiert werden. Insbesondere die Verschreibungen von NOAK stiegen stark an (0,1 % in 2011 auf 5,1 % in 2016) bei gleichzeitiger leichter Abnahme der Verordnungen von Vitamin-K-Antagonisten (7,7 % in 2007 auf 7,1 % in 2016). Diese Ergebnisse passen zu der starken Zunahme des Verschreibungsvolumens von NOAK (von ca. 40 Mio. DDD 2012 auf 330 Mio. DDD in 2016) bei gleichzeitiger Abnahme der Verschreibung von Vitamin-K-Antagonisten (von 330 Mio. DDD in 2008 auf 320 Mio. DDD in 2016; [11–13]), die in Deutschland in den letzten Jahren beobachtet wurde. Hier lässt sich eine Tendenz der Zunahme von Epistaxisfällen unter gleichzeitig erhöhter Häufigkeit der Verschreibung von NOAK beobachten. Die schweren, hospitalisationsbedürftigen Epistaxisfälle scheinen jedoch nicht zuzunehmen.

### Schweregrad und Therapieerfolg

Die hohe Zahl an Patienten, die sich während des gesamten Studienzeitraums nur einmalig aufgrund einer Epistaxis vorstellten, lässt einen hohen Anteil spontaner Selbstheilung vermuten. Eine stationäre Behandlung war nur in 2,0 % aller Epistaxisfälle erfolgt, was vereinbar ist mit den Ergebnissen früherer Studien [5, 28].

### Stärken und Limitationen

Routinedaten stellen eine sehr gut geeignete Datengrundlage für die Untersuchung der vorliegenden Forschungsfrage dar und bieten einige Vorteile gegenüber Primärdaten. Hervorzuheben ist in diesem Zusammenhang die Größe der untersuchten Studienpopulation, welche auch die Untersuchung von eher seltenen Ereignissen ermöglicht. Ein Recall-Bias wird anders als bei einigen Primärdatenerhebungen vermieden. Aufgrund des Vorliegens eines exakten Verschreibungsdatums ist das Risiko einer Fehlklassifikation bezüglich der Arzneimittelverordnungen gering. Andererseits ist es nicht möglich, einen direkten Zusammenhang zwischen Arzneimittelverordnung und einer Diagnose herzustellen, da diese aus abrechnungstechnischen Gründen nur quartalsweise vorliegt. Darüber hinaus gibt es keine Sicherheit dafür, dass die Versicherten die verordneten Arzneimittel auch tatsächlich angewendet haben. Darüber hinaus ist zu beachten, dass sich die Versichertenkollektive unterschiedlicher gesetzlicher Krankenversicherungen unter anderem in Bezug auf den sozioökonomischen Status, das Geschlecht oder auch das Alter unterscheiden [29, 30], was die Generalisierbarkeit der Ergebnisse einschränkt. Letztendlich beinhaltet die Datengrundlage einige Informationen nicht, die wünschenswert gewesen wären, wie Lifestylefaktoren, Over-the-counter(OTC)-Medikamente oder Laborparameter.

Ob Letztere und/oder das höhere Alter für die vergleichsweise häufigere stationäre Behandlungsindikation bei den älteren Patienten verantwortlich war, kann aufgrund der Limitation der Sekundärdatenanalyse nicht kausal beantwortet werden. Auch über die Gründe der beobachteten Zunahme der Epistaxis bei Kindern und Jugendlichen kann nur spekuliert werden (z. B. Zunahme der auslösenden Faktoren oder niederschwellige Vorstellungsrate aufgrund zunehmender Verunsicherung der Eltern). Dies sollte im Rahmen weiterer Studien untersucht werden.

*Zusammenfassend* gibt die vorliegende Sekundärdatenanalyse eine Übersicht über die Häufigkeit und die Inanspruchnahme ärztlicher Leistungen im ambulanten und stationären Sektor. Neben der arteriellen Hypertonie, dem männlichen Geschlecht sowie der typischen Altersverteilung zeigte sich, dass bei Epistaxisfällen auch häufig eine Medikation mit Antikoagulanzien bestand. Außerdem konnte in dieser Studie eine Tendenz der Zunahme von ambulant auftretender Epistaxis bei gleichzeitig ansteigender Verschreibung von NOAK gezeigt werden. Die schweren, hospitalisationsbedürftigen Epistaxisfälle scheinen hier jedoch nicht zuzunehmen. Eine abschließende Beurteilung hinsichtlich eines möglichen kausalen Zusammenhangs insbesondere mit der simultanen Verordnung von Antikoagulanzien muss in weiteren, prospektiven Studien untersucht werden. Diesbezüglich sollte auch die Zunahme bei den beiden jüngsten Patientengruppen weiter untersucht werden, die aufgrund der Limitationen einer Sekundärdatenanalyse nicht geklärt werden konnte.

Angesichts der demografischen Entwicklung und des damit verbundenen Anstiegs der Multimorbidität ist mit einer weiteren Zunahme der Epistaxis zu rechnen.

## Fazit für die Praxis

- Epistaxis ist ein häufiges Symptom in der Arztpraxis.
- Neben der arteriellen Hypertonie, dem männlichen Geschlecht sowie der typischen Altersverteilung zeigte sich, dass bei Epistaxisfällen auch häufig eine Medikation mit Antikoagulanzien bestand.
- Es zeigte sich eine allgemeine Zunahme der Epistaxisprävalenz über den Studienzeitraum, wenn auch kein Anstieg von schweren hospitalisationsbedürftigen Epistaxisfällen.
